# Skin-infiltrating T cells display distinct inflammatory signatures in lichen planus, bullous pemphigoid and pemphigus vulgaris

**DOI:** 10.3389/fimmu.2023.1203776

**Published:** 2023-06-20

**Authors:** Jona Schinner, Tomas Cunha, Johannes U. Mayer, Stefan Hörster, Peter Kind, Dario Didona, Corinna Keber, Michael Hertl, Thomas Worzfeld, Hazem A. Juratli

**Affiliations:** ^1^ Institute of Pharmacology, Philipps-Universität Marburg, Marburg, Germany; ^2^ Department of Dermatology and Allergology, Philipps-Universität Marburg, Marburg, Germany; ^3^ Center for Dermatopathology, Freiburg, Germany; ^4^ Laboratory for Dermatohistology, Offenbach am Main, Germany; ^5^ Institute of Pathology, Philipps-Universität Marburg, Marburg, Germany; ^6^ Department of Pharmacology, Max-Planck-Institute for Heart and Lung Research, Bad Nauheim, Germany; ^7^ Department of Dermatology, University Hospital Basel, Basel, Switzerland; ^8^ Pathology Unit, University Hospital Basel, Basel, Switzerland

**Keywords:** lichen planus, bullous pemphigoid, pemphigus vulgaris, T cells, immunological signature, Th1/Th2, IL-17, blistering disorders

## Abstract

**Introduction:**

We here thought to dissect the inflammatory signature in lesions of three skin disorders, which show a common adaptive immune response against autoantigens of the skin but are characterized by diverging clinical phenotypes. Pemphigus vulgaris (PV) and bullous pemphigoid (BP) are type-2-dependent, IgG autoantibody-driven blistering disorders of mucous membranes and skin, which target desmoglein (Dsg)3 and bullous pemphigoid (BP)180, respectively. In contrast, lichen planus (LP) is a common chronic inflammatory disease of the skin and mucous membranes with a pronounced dermal T cell infiltrate. We previously identified peripheral type 1 and 17 T cell responses against Dsg3 and BP180 in a cohort of LP patients strongly suggesting that the underlying inflammatory T cell signature may drive the evolving phenotype.

**Methods:**

Paraffin-embedded skin biopsies from well-characterized patients with LP (n=31), BP (n=19), PV (n=9), and pemphigus foliaceus (PF) (n=2) were analysed. Areas with the most prominent inflammatory infiltrate were excised with punch biopsies and tissue microarrays (TMA) containing multiple biopsies were created. Using multicolor immunofluorescence, the inflammatory infiltrate was stained with antibodies against multiple cellular markers, i. e. CD3ϵ, CD4, CD15, TCR-δ, the cytokine IL-17A, and the transcription factors, T-bet and GATA-3.

**Results:**

In LP, there was a higher number of CD4+ T cells expressing T-bet compared to GATA-3. In contrast, CD4+ T cells in PV and BP skin lesions more frequently expressed GATA-3 than T-bet. IL-17A+ cells and IL-17A+ T cells were found to a similar extent in all the three disorders. IL-17A+ granulocytes were more predominant in BP than in LP or PV. Of note, the majority of IL-17A+ cells in LP were neither T cells nor granulocytes.

**Discussion:**

Our findings in inflammatory skin infiltrates clearly show a predominant type 1 signature in LP in contrast to a preponderance of type 2 T cells in PV and BP. In contrast to LP, granulocytes and to a much lesser extent CD3+ T cells were a cellular source of IL-17A in BP and PV. These data strongly suggest that different inflammatory cell signatures drive evolving clinically diverse phenotypes of LP, PV and BP despite common target antigens of the skin.

## Introduction

Chronic inflammatory skin disorders associated with autoimmune responses remain poorly understood, due to a lack of knowledge about their specific target antigens, disease variability and co-morbidities. Immunologically, they can be grouped into disorders associated with T cell-mediated and with antibody-mediated autoimmune responses, both of which require the activation of autoreactive T cell subsets against target proteins expressed in the epidermis and dermis.

Lichen planus (LP) is one of the most common T cell-mediated autoimmune diseases of the skin and mucous membranes, and affects 1-2% of the general population (1–3). Clinically, it presents as reticular, purple papules and plaques of the skin and white papules and erosions of mucous membranes, and follows a chronic-relapsing course (4–6). LP mucosal and skin lesions are infiltrated by T cells which include CD8+ and CD4+ populations. Most relevant for the direct pathology of LP are pro-inflammatory CD8+ T cells, which are mainly located around the basal layer of the epidermis where they trigger apoptosis of epidermal keratinocytes (1, 2, 6–8). While the targeted autoantigens have not been fully identified, self-peptides of keratinocyte-derived proteins presented on HLA class I and II alleles may play a central role in disease pathogenesis (4, 7). Several type 1 and recently type 17 cytokines such as IFN-γ, TNF-α , IL-17A and IL-22 have been detected in LP lesions, as well as in patient sera, supporting the concept that LP is a highly inflammatory skin disorder (8, 9). These cytokines are thought to be mainly produced by CD8+ T cells, and to a lesser extent, by CD4+ T helper cell populations (10), which display a Th1 and Th17 phenotype. While their recruitment and activation mechanisms remain to be elucidated, pro-inflammatory cytokines produced by these populations play an important role in disease pathogenesis, and initial studies using small molecules or anti-cytokine monoclonal antibodies have shown promising results (11–14).

Autoantibody-mediated autoimmune diseases, such as pemphigus vulgaris (PV) and bullous pemphigoid (BP), share clinical similarities with mucosal LP. PV and BP are characterized by blisters and erosions of the skin and mucous membranes, which are caused by IgG autoantibodies against distinct adhesion proteins of desmosomes and hemidesmosomes. In PV and pemphigus foliaceus (PF), IgG autoantibodies are directed against components of desmosomes, namely desmoglein (Dsg) 3 and Dsg1. In BP, IgG targeting hemidesmosomal antigens, namely bullous pemphigoid antigen 1 (BP230) and bullous pemphigoid antigen 2 (BP180) induce the influx of innate immune cells in the skin leading to pronounced inflammation with pruritus and tense blisters.

The cellular infiltrate in BP and PV skin lesions is very heterogeneous and consists primarily of neutrophilic granulocytes (BP), eosinophils, CD4+ T cells and B lymphocytes, yet is less inflammatory than LP. Autoreactive T and B lymphocytes play a central role in the pathogenesis of both, PV and BP, and pro-inflammatory autoreactive Th2 cells are observed at a higher ratio in PV and BP than in LP (15).

A recent single-cell RNA-sequencing study in a small number of LP and BP patients showed that both diseases lack infiltrating myeloid derived populations, when compared to healthy controls (HC), atopic dermatitis or psoriasis lesions, yet display distinct T cell signatures (16). This correlates with our own recent findings which showed that patients with LP exhibit IFN-γ- and IL-17A-dominated peripheral blood T cell responses, while BP patients display a higher number of peripheral type 2 autoimmune T cell responses (15). In LP, BP180-specific peripheral Th17 cell responses correlated with a distinct Th17 cell infiltrate along the dermal-epidermal basement membrane zone in LP skin lesions. This observation strongly suggests that IL-17A is a major driver of disease pathogenesis. IL-17A polymorphism and increased serum levels of IL-17A are indeed associated with LP (17, 18), not only affecting local infiltration, but also the microbiome of oral LP patients (19). Therapeutic targeting of IL-17 or IL-23 (which is involved in Th17 development) by monoclonal antibodies has also shown positive effects in several case series (12, 14), suggesting that IL-17A producing CD4+ or CD8+ T cells play an important role in LP.

In light of the above observations, we hypothesized that LP, BP, and PV are inflammatory disorders which target identical autoantigens of the skin although with a different T cell signature. This led previous research to study whether functional differences of the evolving inflammatory skin infiltrate may eventually pave the way for the distinct clinical phenotypes that we see in LP, PV and BP in humans. A previous study has shown that mice with a transgenic T cell receptor for desmoglein 3 develop a type 1 inflammatory skin infiltrate resulting in a lichenoid phenotype with a dense band-like lymphocytic infiltrate resembling LP (20). These findings are in support of our initial hypothesis that LP is a type 1 variant of PV and BP, respectively. So far, thorough characterization of the inflammatory T cell responses in these disorders was mainly performed with peripheral blood lymphocytes. To characterize the immune signature of skin-infiltrating cells in more detail, we analyzed key transcription factors involved in type 1 and type 2 cell development, as well as IL-17A production by T cells and granulocytes in PV, BP and LP on tissue microarrays (TMA) of skin lesions using immunofluorescent antibody staining. Our findings link LP to a type 1-regulated inflammatory skin pathology while skin lesions from PV and BP patients show a more pronounced type 2 CD4+ T cell response.

## Results

### Dichotomy of type 1 CD4+ T cells in LP versus type 2 T cells in BP and PV

**Figure 1:**
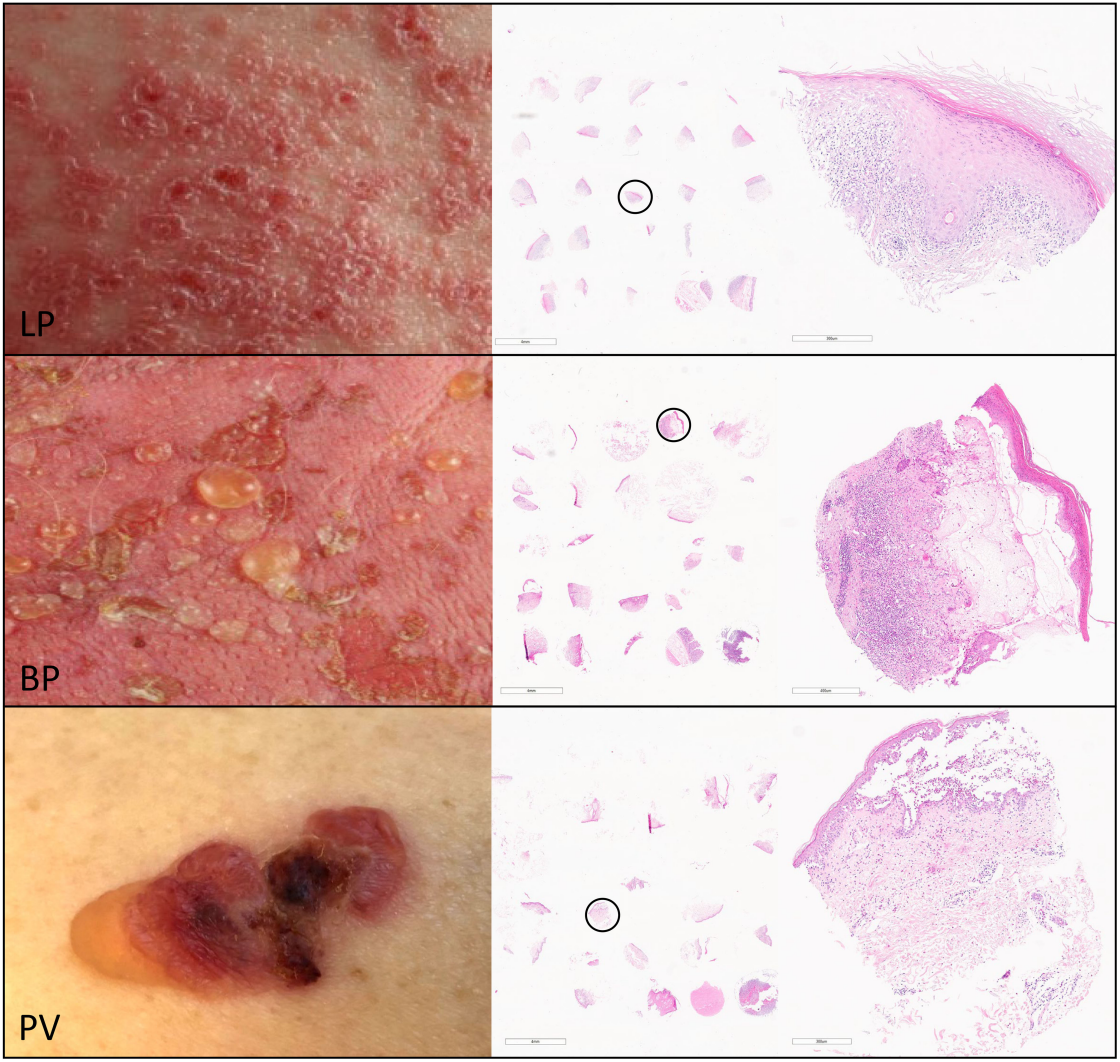
For the characterization of the inflammatory infiltrate of skin lesions in lichen planus (LP), bullous pemphigoid (BP) and pemphigus vulgaris (PV) we generated tissue micro arrays (TMAs) using punch biopsies from the inflammatory epidermal/dermal infiltrate of paraffin-embedded skin lesions from patients with LP (upper panel), BP (middle panel) and PV (lower panel). The right column shows representative hematoxylin-eosin staining of TMA sections from LP, BP and PV with a magnification of the circled punch biopsy.

To characterize the immune signatures of the inflammatory CD4+ skin infiltrate in LP, BP, and pemphigus patients we collected paraffin-embedded skin biopsies. In total, the cohort comprised samples from 31 LP, 19 BP and 11 pemphigus patients. Within the pemphigus group, both PV (n=9) and pemphigus foliaceus (PF; n=2) were included. However, for statistical analyses, the PF samples were omitted due to the low sample number. All paraffin-embedded skin biopsies were re-embedded to generate tissue microarrays (TMAs) (**Figure 1**). The TMAs were then subjected to immunostaining with antibodies against distinct immune cell subsets.

**Figure 2:**
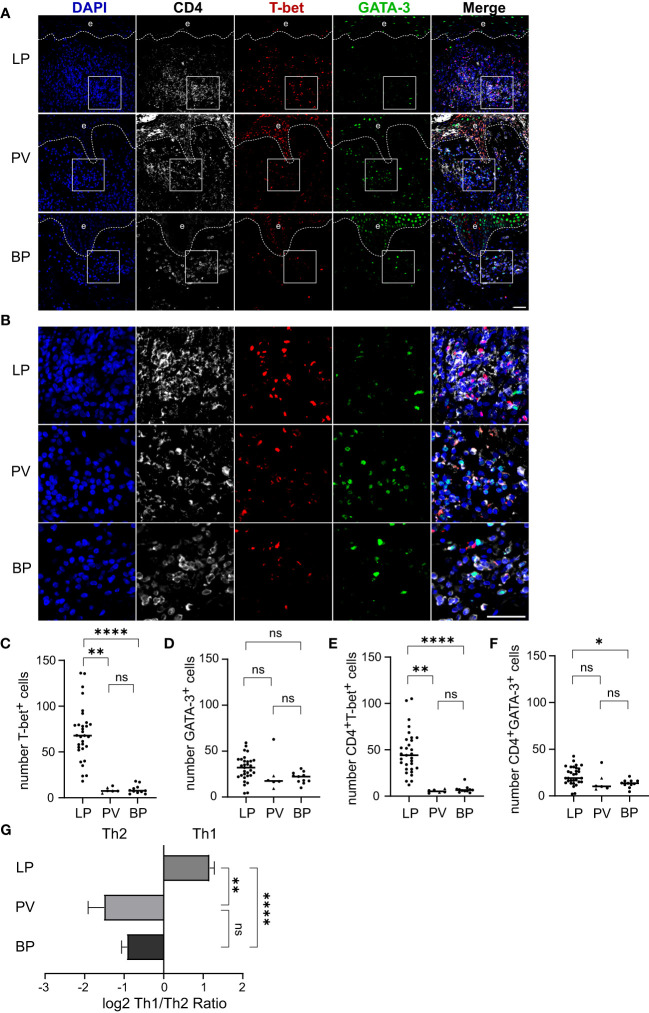
Th1/Th2 ratio in dermal immune cell infiltrates of LP, PV and BP patients. **(A, B)** Representative confocal images of immunostaining of tissue microarrays using anti-CD4 (white), T-bet (red) and GATA-3 (green) antibodies. Dashed lines in **(A)** indicate the border between epidermis **(e)** and dermis. Boxed areas in **(A)** are magnified in **(B)**. Blue: 4′,6-diamidino-2-phenylindole (DAPI). Scale bars represent 40 µm. **(C–F)** Quantification of **(C)** T-bet-positive, **(D)** GATA-3-positive, **(E)** CD4-/T-bet-double-positive and **(F)** CD4-/GATA-3-double-positive cells per tissue section. Horizontal bars indicate median values and individual points represent a biopsy from a different patient with LP (n = 31), PV (n = 4) and BP (n = 11). Triangles in the PV-Group represent biopsies from patients with PF (n = 2), which were excluded from statistical analysis. **(G)** Quantification of the log2 transformed ratio between CD4-/T-bet-double-positive (Th1) and CD4-/GATA-3-double-positive (Th2) cells. Shown are mean values + S.E.M. Statistical significance was assessed using Kruskal-Wallis test with Dunn’s correction for multiple comparison. *p ≤ 0.05, **p ≤ 0.01, and ****p ≤ 0.0001. n.s., not significant.

It is known that CD4+ T helper (Th) cells play a pivotal role in inflammatory skin disorders. However, the predominant differentiation signatures of CD4+ T helper cells in the skin infiltrates in LP, BP and PV remain unclear. We therefore aimed at the visualization and quantification of Th1 and Th2 cells. To do so, we performed co-immunostaining for the Th cell marker CD4 together with T-bet and GATA-3, the master transcription factors for Th1 and Th2 cells, respectively (**Figures 2A–D**). In LP we found a significantly higher number of both CD4+/T-bet+ cells as well as CD4+/GATA-3+ cells compared to BP with a median of 44 CD4+/T-bet+ cells in LP and a median of 6.5 cells in BP (p < 0.0001) and a median of 19 CD4+/GATA-3+ cells in LP compared to a median of 13.5 cells in BP (p = 0.025). There was a significantly higher median of 44 CD4+/T-bet+ cells in LP compared to 5.2 cells in PV (p = 0.001) and no significant differences between BP and PV for both populations were observed (**Figures 2E, F**). Of note, Th1/Th2 cell ratios were highly significantly different between the three groups of LP, PV and BP patients: While LP skin lesions showed a preponderance of Th1 cells, PV and BP skin lesions predominantly contained Th2 cells (**Figure 2G**).

### Type 17 T cells in skin lesions from LP, BP and PV/PF patients

**Figure 3:**
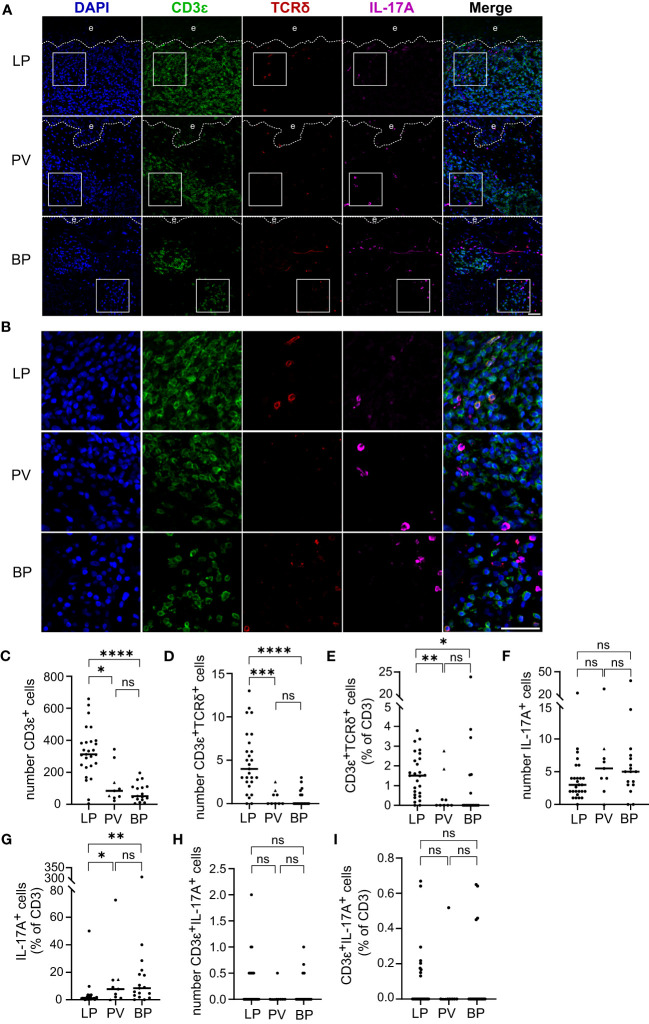
T cell infiltrate, γδ T cell proportion and IL-17A producing cells in LP, PV and BP patients. **(A, B)** Representative confocal images of immunostaining of tissue microarrays using anti-CD3ϵ (green), TCRδ (red) and IL-17A (purple) antibodies. Dashed lines in **(A)** indicate the border between epidermis **(e)** and dermis. Boxed areas in **(A)** are magnified in **(B)**. Blue: 4′,6-diamidino-2-phenylindole (DAPI). Scale bars represent 40 µm. **(C–E)** Quantification of **(C)** CD3ϵ-positive cells per tissue section, **(D)** CD3ϵ-/TCRδ-double-positive cells per tissue section and **(E)** proportion of CD3ϵ-/TCRδ-double-positive cells as percentage of total CD3ϵ-positive cells. **(F, G)** Quantification of **(F)** IL-17A-positive cells per tissue section and **(G)** IL-17A-positive cells compared to the CD3ϵ-positive cells expressed as percentage. **(H, I)** Quantification of **(H)** CD3ϵ-/IL-17A-double-positive cells per tissue section and **(I)** fraction of CD3ϵ-/IL-17A-double-positive cells as percentage of total CD3ϵ-positive cells. Horizontal bars indicate median values and individual points represent a biopsy from a different patient with LP (n = 27), PV (n = 7) and BP (n = 17). Triangles in the PV-Group represent biopsies from patients with PF (n = 2), which were excluded from statistical analysis. Statistical significance was assessed using Kruskal-Wallis test with Dunn’s correction for multiple comparison. *p ≤ 0.05, **p ≤ 0.01, ***p ≤ 0.001, and ****p ≤ 0.0001. n.s., not significant.

In addition to Th1 and Th2 cells, IL-17 producing T cells have been shown to be of major pathophysiological relevance in inflammatory skin diseases (21, 22). Whether T cell receptor (TCR)-α/β+ T cells or TCR-γ/δ+ T cells serve as IL-17A producers in the inflammatory skin infiltrate of LP, BP and/or PV is ill-defined. We therefore immunostained skin lesions for CD3ε (CD3 as a pan-T cell marker), TCR-δ chain (a marker for γδ T cells), and IL-17A (**Figures 3A, B**). As expected, the highest median of 312 CD3ε+ cells was found in LP skin lesions with significant differences compared to the median of 84 CD3ϵ+ cells in PV (p = 0.03) and 51 CD3ϵ+ cells in BP (p < 0.0001) (**Figure 3C**). In LP a median of 1.5% of the total T cell infiltrate were γδ T cells which was a significantly higher fraction compared to the median of 0% in both PV (p = 0.004) and BP (p = 0.02) (**Figures 3D, E**).

In the analyzed skin disorders, IL-17A+ cells were observed in similar amounts, with a median of 3 IL-17A+ cells in LP, 5.5 in PV, and 5 in BP (**Figure 3F**). However, when normalized to the CD3ϵ+ infiltrate, there were significantly more IL-17A+ cells in PV (p = 0.02) and BP (p = 0.001) compared to LP (**Figure 3G**). Only a few CD3ϵ+/IL-17A+ cells were found, with no significant differences in both the absolute numbers and fraction of total T cells (**Figures 3H, I**). It is noteworthy that most IL-17A+ cells were negative for CD3ϵ, indicating that they were not T cells (compare **Figures 3F, H**), and none of the identified γδ T cells expressed IL-17A (data not shown). This prompted us to study inflammatory cells other than T cells as a potential source of IL-17A.

### IL-17-producing granulocytes are predominant in BP

**Figure 4:**
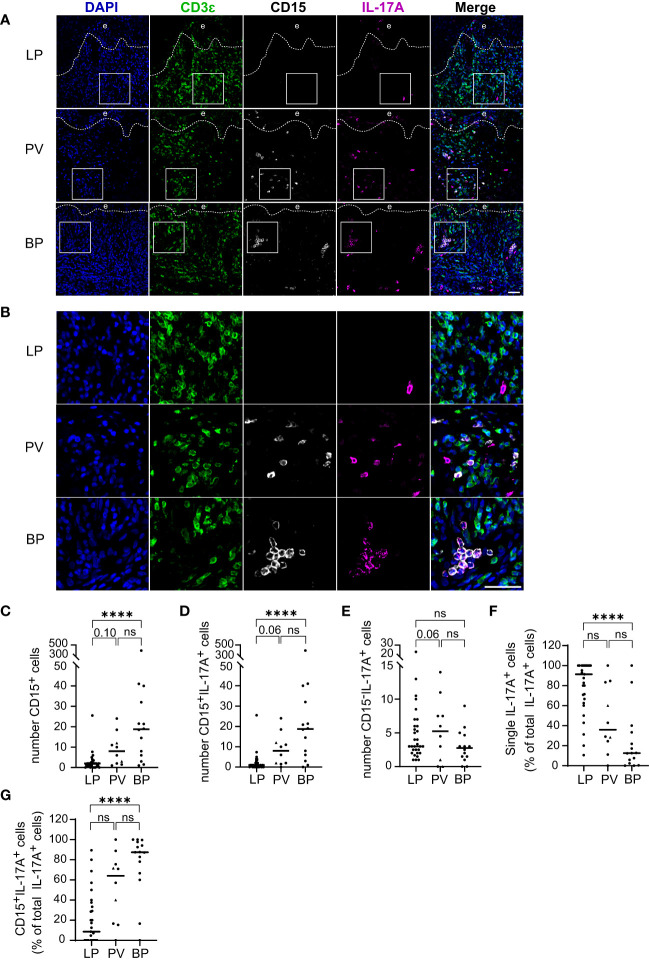
IL-17A producing Granulocytes in LP, PV and BP patients. **(A, B)** Representative confocal images of immunostaining of tissue microarrays using anti-CD3ϵ (green), CD15 (gray) and IL-17A (purple) antibodies. Dashed lines in **(A)** indicate the border between epidermis **(e)** and dermis. Boxed areas in **(A)** are magnified in **(B)**. Blue: 4′,6-diamidino-2-phenylindole (DAPI). Scale bars represent 40 µm. **(C–E)** Quantification of **(C)** CD15-positive **(D)** CD15-/IL-17A-double-positive and **(E)** IL-17A-positive/CD15-negative cells per tissue section. **(F, G)** Quantification of the proportion of **(F)** single IL-17A-positive (CD3ϵ-negative/CD15-negative) cells and **(G)** CD15-/IL-17A-double-positive cells as percentage of total IL-17A-positive cells. Horizontal bars indicate median values and individual points represent a biopsy from a different patient with LP (n = 31), PV (n = 8) and BP (n = 14). Triangles in the PV-Group represent biopsies from patients with PF (n = 2), which were excluded from statistical analysis. Statistical significance was assessed using Kruskal-Wallis test with Dunn’s correction for multiple comparison. ****p ≤ 0.0001. n.s., not significant.

Our finding that the majority of IL-17A+ cells are no T cells prompted us to investigate the identity of the inflammatory cell populations other than T cells expressing IL-17A. Given that neutrophils, the most abundant of the granulocytes, are a common source of IL-17, we focused on CD15+ granulocytes in the skin infiltrates of LP, BP, and PV patients (**Figures 4A, B**). The total number of CD15+ granulocytes and CD15+/IL-17A+ cells were significantly greater in BP patients than in LP patients (**Figures 4C, D**). The median number of IL-17A+ granulocytes was 18.75 in BP and only 1 in LP (p < 0.0001). There was also a trend towards a higher median of granulocytes in PV compared to LP (**Figures 4C, D**). The medians of IL-17A+ cells that were negative for CD15 did not differ significantly between the three groups (**Figure 4E**). In LP, the majority of IL-17A+ cells were neither granulocytes nor T cells, whereas in BP and PV, most IL-17A+ cells were CD15+ granulocytes (**Figures 4F, G**).

### Monoclonal antibodies against IL-17 ameliorate skin lesions in lichen planus

**Figure 5:**
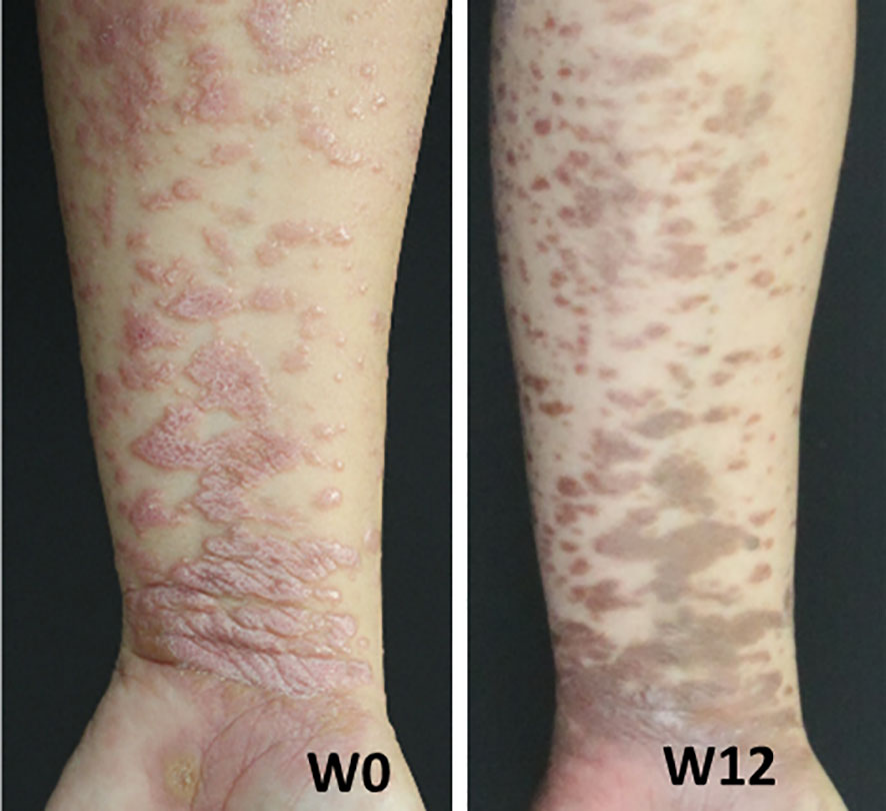
Lichen planus patient was treated with the anti-IL-17A monoclonal antibody Secukinumab at 300 mg administered weekly for 5 weeks starting at week 0, followed by monthly doses. Shown are images of the patient's left forearm at week 0 (W0) and week 12 (W12).

To gain insight into the functional consequences of the distinct inflammatory signatures in LP, we studied the clinical effect of therapeutic cytokine inhibition in a patient with LP. As the majority of skin-derived IL-17A+ cells in LP neither belong to T cells nor to granulocytes, we sought to determine the functional impact of IL-17A+ cells on LP pathogenesis in general. In a 28-year-old female patient with extensive cutaneous LP, we took advantage of the availability of Secukinumab, an anti-IL-17A monoclonal antibody which is approved for the treatment of psoriasis vulgaris. The LP patient was not sufficiently responsive to topical steroids and was treated in a compassionate trial with Secukinumab at 300 mg s.c. weekly for four weeks and then 300 mg every four weeks. At week 12 of treatment with Secukinumab, the initially reddish erythematous plaques had completely disappeared leaving brownish hyperpigmentations (**Figure 5**).

## Discussion

The present study provides a comprehensive analysis of the inflammatory signature of skin lesions in three inflammatory skin disorders linked to T cell recognition of well-known autoantigens of the skin and mucous membranes. PV/PF and BP are IgG-driven autoimmune bullous skin disorders targeting the cutaneous autoantigens, Dsg3/Dsg1 and BP180, respectively. Previous studies by our group and others had identified mainly type 2 peripheral blood autoreactive T cells in both disorders (23–26). In contrast, we had identified type 1 and type 17 peripheral blood T cells reactive with Dsg3 and BP180 in patients with mucosal and cutaneous LP (15). As these studies have mainly focused on the peripheral blood, we here sought to investigate whether these peripheral signatures correspond to the signatures within the skin lesions that have remained largely unknown.

The major aim of the present study was therefore to study skin sections from a cohort of PV, BP and LP patients in order to investigate whether a similar polarized CD4+ T cellular signature could be identified in lesions of these inflammatory skin disorders. We generated TMAs allowing us to perform a comparative analysis of skin infiltrates with greater patient numbers and small inter-assay variability. Simultaneous staining of TMA containing several skin sections provides a practicable and suitable research instrument for immune profiling studies. Based on the ratio of CD4+/T-bet+ (Th1) and CD4+/GATA-3+ (Th2) cells, we clearly identified a type 1 inflammatory signature in LP skin lesions in contrast to a predominant type 2 inflammatory signature in BP and PV skin lesions.

It is well-known that LP lesions are infiltrated by large numbers of CD8+ and CD4+ T cells with a pro-inflammatory signature (4). Cytokines produced by these cells include IFN-γ, TNF-α, IL-6, IL-17, IL-22, which are not only observed in skin lesions but can also be detected in patients` sera (9, 27). While cytotoxic CD8+ T cells are presumably the main drivers of disease and target basal keratinocytes, CD4+ T cells produce the majority of proinflammatory cytokines. The spectrum of (auto)antigens which are recognized by these T cells and how they are recruited to LP skin lesions remains to be elucidated. Candidate target antigens of epidermal keratinocytes and components of the dermal-epidermal junction, such as Dsg3, BP180 and others including microbial peptides have been recently identified (19, 28). It is also possible that these cytokines play an important role in disease progression, as proinflammatory cytokines reduce wound healing, impact peripheral tolerance and enhance blister formation (10).

In contrast to LP, T cell infiltrates are much less pronounced in BP and PV skin lesions as confirmed in the present study and already strongly suggested by previous work (15, 29, 30). This is primarily linked to the distinct immunopathology associated with these diseases, as BP and PV are driven by autoantibodies whereas LP is mainly associated with effector T cells. While it is hard to identify autoreactive B cells in BP and PV skin lesions, the draining lymph nodes or the bone marrow, IgG autoantibodies can readily be detected in patients’ sera, indicating their systemic distribution (31).

Autoantibody-mediated acantholysis in PV is presumably largely independent from immune cell infiltration and can readily be achieved *in vitro* and *in vivo* in preclinical animal models (32), suggesting that inflammatory immune cells are not directly required for disease progression. In contrast, BP which is characterized by subepidermal loss of adhesion largely depends on complement fixing IgG autoantibodies which lead to an influx of innate immune cells such as neutrophils and macrophages, which secrete proteolytic enzymes eventually leading to blister formation. While BP and PV blisters display a mild to moderate inflammatory phenotype, innate inflammation is not a key driver of disease and presumably more likely a secondary inflammatory response to epidermal cell death, barrier disruption and exposure to infiltrating microbes (33). The T cell signature in BP and PV is therefore less proinflammatory than in LP, and CD8+ T cells are observed at a much lower frequency. A higher ratio of anti-inflammatory GATA-3+ Th2 cells, which are associated with wound healing, barrier homeostasis and B cell activation can also be observed in BP and PV lesions (12, 15). However, type 2 T cells can also induce proinflammatory responses, cause itch, recruit eosinophils, basophils and cause their activation (34). In our opinion, the present approach to directly identify polarized T cell responses, i.e. Th1 versus Th2 versus IL-17A producing T cells extends previous studies looking at cytokine expression in skin lesions from patients with LP, BP and PV (35, 36). In contrast to these previous studies, the present study allows to directly identify and quantitate defined T cell subsets in the skin infiltrate of these inflammatory skin disorders.

It is still a matter of debate as to whether type 2 cells play an anti- or pro-inflammatory role in BP and PV, but treatment with Dupilumab, an anti-IL-4Ra antibody, has shown positive results in a small cohort of BP patients with over 50% of patients showing complete clearance of disease (37). The T cell dichotomy between Th1 and Th2 cells observed in LP vs BP/PV might therefore have important clinical implications for targeted therapies that reduce disease pathogenesis.

In recent years, a distinct T cell population residing in human epithelia, characterized by the expression of IL-17 has received major attention, as these cells may possess pro-inflammatory function thereby promoting the pathology of several skin diseases and chronic inflammatory immune responses (22, 38). IL-17+ Th17 and Tc17 cells have been now identified as suitable therapeutic targets in psoriasis vulgaris and additional inflammatory skin disorders (**Table 1**). In the present study, IL-17A+ cells were found in all three skin disorders to a similar extent. While LP showed a few IL-17A+ T cells, a significant number of IL-17A+ cells were neither T cells nor granulocytes. Of note, γδ T cells, which were more common in LP than in BP or PV are potential IL-17A producers. Skin lesions from patients with BP contained to the greatest extent IL-17A-producing granulocytes. Moreover, PV skin lesions contained IL-17A+ T cells and granulocytes in addition to IL-17A+ cells of unknown origin.

**Table 1:** Interleukin-17 (IL-17) producing cells in human skin.

Cell type	Disease	Location	Publication
**CD3+ CD4+**	Psoriasis Vulgaris	subepithelial layer	10.1046/j.1523-1747.1998. 00347.x
**Th17 cells**			10.1371/journal.pone.0014108
			10.1007/s10875-012-9716-x
			10.1007/s11596-007-0329-1
			10.1038/jid.2008.85
	Atopic Dermatitis	epidermis	10.1038/jid.2008.111
	Allergic contact dermatitis	epidermis	10.1111/j.1365-2133.2009. 09400.x
		subepithelial layer	10.4049/jimmunol.0901767
			10.1111/j.1365-2133.2009. 09400.x
			10.1111/cod.12043
			10.1111/all.12351
	Lichen Planus	subepithelial layer	doi/10.1111/jop.12898
			10.3389/fimmu.2019.01808
	Bullous Pemphigoid	subepithelial layer	10.1016/j.jaut.2018.09.003
			10.1038/srep18001
			10.1155/2013/967987
			10.1111/j.1600-0625.2011.01378.x
	Vitiligo	epidermis	10.1111/j.1365-2230.2010. 03972.x
	Cutaneous lupus erythematosus	epidermis	10.1111/j.1365-2230.2010. 03996. x
	Cutaneous T-Cell Lymphoma	dermis	10.1111/j.1365-2133.2011. 10647.x
	Basal cell carcinoma	epidermis	10.1002/eji.201445052
	Hidradenitis suppurativa	epidermis	10.1111/bjd.14075
	Cutaneous Candidiasis	epidermis	10.1007/s12026-011-8226-x
			10.3390/pathogens4030606
**CD3+ CD8+**	Psoriasis Vulgaris	subepithelial layer	10.1046/j.1523-1747.1998. 00347.x
**Tc17 cells**			10.1371/journal.pone.0014108
			10.1007/s10875-012-9716-x
			10.1007/s11596-007-0329-1
			10.1038/jid.2008.85
	Lichen Planus	epidermis	10.1111/jop.12898
	Atopic Dermatitis	stratum corneum	10.1038/jid.2012.456
	Allergic contact dermatitis	epidermis	10.1111/j.1365-2133.2009. 09400.x
	Graft-Versus-Host Disease	dermis	10.1007/s00005-019-00549-2
**TCR−γ/δ+**	Lichen Planus	epidermis	10.1111/1523-1747.ep12394904
**T cells**	Atopic Dermatitis	epidermis	10.1111/ijd.16364
	Psoriasis Vulgaris	epidermis	10.1016/j.immuni.2011.08.001
			10.1016/j.ebiom.2022.104136
			10.1038/nm.3016
			10.1007/s10875-008-9233-0
**Neutrophilic granulocytes**	Psoriasis Vulgaris	dermis	10.4049/jimmunol.1100123
			10.1111/bjd.15533
	Cutaneous T-Cell Lymphoma	dermis	10.1111/j.1365-2133.2011. 10647.x
	Hidradenitis suppurativa	epidermis	10.1111/bjd.14214
	Bullous pemphigoid	dermis	10.1038/jid.2014.263

Similarly to LP and BP, IL-17A+ T cells were present in skin lesions from PV patients which confirms previous findings (39). Still, their pro- or anti-inflammatory function remains to be elucidated. It is noteworthy that genetic deficiency of Dsg1, the autoantigen of PF, leads to a Th17 signature as do antibodies against Dsg1 (40). Th17 cells and related cytokines are expressed in PV/PF and BP skin lesions. Their direct impact on the immune pathogenesis, e.g. the local production of IgG autoantibodies, has not yet been demonstrated (41, 42). In contrast, the majority of IL-17+ inflammatory cells in LP skin lesions do not belong to the T cell or granulocyte compartment. This finding is novel and of potential pathophysiological importance since monoclonal antibodies against IL-17 have been shown to significantly improve LP skin lesions (15). Finally, the third cellular source of IL-17 are neutrophilic granulocytes, which are a major inflammatory cell subset in BP skin lesions. IL-17 dependent activation of IL-1ß associated inflammasome augments the innate autoinflammatory immune responses inherent to BP (43). Moreover, blockade of IL-17-induced neutrophil recruitment into BP skin leads to amelioration of skin symptoms (44).

In LP, serum levels of IL-17 were readily detectable and IL-17+ CD4+ and CD8+ T cells were observed in LP lesions (8, 17). A recent meta-analysis including more than 600 patients with oral LP (OLP) observed that increased serum levels of IL-17 are strongly associated with the clinical disease severity of LP (45). Under inflammatory conditions, IL-17 induced chemokines lead to the recruitment of pro-inflammatory effector cells in the skin and the proliferation of keratinocytes (22). Keratinocyte proliferation might further increase the pathological autoantigen pool in LP, leading to further disease progression. While the major producers of IL-17 include CD4+ Th17 cells, which express the transcription factor ROR-γt, other IL-17 immune cell populations have also been identified in inflamed skin (see **Table 1**). These include IL-17-producing CD8+ T cells, also known as Tc17 cells, γδ T cells, as well as IL-17+ neutrophilic granulocytes, all of which infiltrate the epidermis or the subepithelial layer. While the development of Th17 cells requires IL-23 signaling, which is produced by dendritic cells during T cell priming, it is unclear which factors trigger the production of IL-17 in other cell types (22). Several monoclonal antibodies against IL-17, as well as IL-23, have been developed and have been shown to improve inflammatory skin diseases such as psoriasis (22). Individual LP patients have already been treated with monoclonal antibodies against IL-17 and IL-23 (12, 14), which has resulted in a clinical as well as a histological improvement of disease. Shown here is a patient with extensive LP of the skin who showed an excellent therapeutic response, i.e. fast regression of skin lesions upon treatment with the IL-17A blocker Secukinumab. However, a larger cohort size and controlled setting as part of a clinical trial will be necessary to determine the efficacy of IL-17 blockade in LP and its clinical variants.

In summary, our findings support the concept that LP is a type 1 T cell-associated skin disorder with a type 17 component while PV and BP show type 2 cells with a strong autoantibody-driven pathogenesis. These findings are in line with the characterization of peripheral blood type 1/type 17 signatures in LP and a type 2 T cell response in PV. These immunological signatures may pave the way for the treatment of LP using antibodies targeting Th1-associated cytokines, as well as for PV and BP using antibodies targeting Th2-associated cytokines.

## Material and methods

### Human subjects

Formalin-fixed paraffin-embedded (FFPE) skin tissues from patients with LP (n=31), BP (n =19), PV (n=9) and PF (n=2) were included in the study. The cohorts were kindly provided by the Center for Dermatopathology in Freiburg (S. Hörster) and Dermatopathology (P. Kind) in Offenbach (**Supplementary Table 1**). The samples were processed, pseudonymized and included in the present study based on their diagnoses. The LP samples were included based on histologic criteria and the BP and PV samples were included based on histologic, direct immunofluorescence and immune serological criteria. The disease classification criteria were as follows:

1. Lichen planus: clinical suspicion (polygonal reddish papules on the skin) and histopathology showing a band-like subepidermal lymphocytic infiltrate in the upper dermis and sawtoothed rete ridges.
2. Bullous pemphigoid: clinical suspicion (tense blisters/erosions or polymorphic pruritic lesions of the skin); histopathology: subepidermal loss of adhesion and DIF: IgG and/or C3 at the dermal-epidermal junction or IIF: anti-basement membrane serum antibodies on monkey esophagus or ELISA: anti-BP180/BP230 serum IgG
3. Pemphigus vulgaris/foliaceus: clinical suspicion (flaccid blisters/erosions of the oral mucosa and/or skin); histopathology: intraepidermal loss of adhesion and DIF: IgG and/or C3 on the surface of epidermal keratinocytes or IIF: anti-epithelial serum antibodies on monkey esophagus or ELISA: anti-desmoglein 3/desmoglein 1 serum IgG.
This study was approved by the Ethics Committee of the Medical Faculty, Philipps University Marburg (165/19).

### Tissue microarrays

To generate tissue microarrays (TMA), 2 mm punch biopsies were taken from FFPE tissues utilizing a Manual tissue arrayer (MTA-1) from Alphametrix Biotech, within regions of infiltrating cells. The excised tissues were combined and re-embedded in a newly arrayed recipient paraffin block, according to standard procedures of the Comprehensive Biomaterial Bank Marburg (CBBM).

### Immunofluorescence staining

4 µm sections of TMAs were deparaffinized and rehydrated by two 5 min immersions in Xylene followed by 5 min in 99% ethanol and consecutive immersions in 95%-, 80%- and 70%-ethanol for 3 min each. After two 3 min washes in ddH_2_O heat-induced antigen retrieval was performed in 10 mM Citrate Buffer pH 6.0 for 10 min using a pressure cocker. After 45 min cool down and a following 1 min wash in ddH_2_O the sections were permeabilized by immersion in 0.2% Triton X-100 in PBS (PBST) for 10 min. Sections were blocked with 10% FCS in PBST for 1 h, primary antibodies were added in 2% FCS in PBST and incubated over night at 4°C in a humidified chamber. On the next day sections were washed three times with PBS for 10 min each followed by 2 h incubation with secondary antibodies in 1.5% FCS in PBST together with 4′,6-Diamidin-2-phenylindol (DAPI) in a humidified chamber at room temperature. After three consecutive washes in PBS the slides were mounted with Dako fluorescence mounting medium. The primary and secondary antibodies used as well as the dilutions can be found in **Supplementary Table 2**.

### Image acquisition and cell counting

A Zeiss LSM 700 confocal microscope with a 20x objective was used to acquire 320 µm x 320 µm images of the immune cell infiltrate at the dermal-epidermal junction. The immunofluorescence images were analyzed using ImageJ (v 1.53c). Positively stained cells were manually counted when CD3ϵ, TCR-δ, CD4 or CD15 showed signals at the cell membrane around the DAPI+ nucleus. Since T-bet and GATA-3 are transcription factors positive cells were counted when the signal showed an overlap with DAPI. Cells were considered IL-17A positive when the signal was between the nucleus and the cell membrane.

### Statistical analysis

Kruskal-Wallis test with Dunn’s correction for multiple comparison was used to test for statistical significance between groups. Level of significance is indicated by asterixis with *p ≤ 0.05, **p ≤ 0.01, ***p ≤ 0.001, and ****p ≤ 0.0001, whereas ns stands for not significant. The analysis was performed using GraphPad Prism Version 9.5.0.

## Data availability statement

The raw data supporting the conclusions of this article will be made available by the authors, without undue reservation.

## Ethics statement

The studies involving human participants were reviewed and approved by Ethics Committee of the Medical Faculty, Philipps University Marburg (165/19). Written informed consent for participation was not required for this study in accordance with the national legislation and the institutional requirements. Written informed consent was obtained from the individual(s) for the publication of any potentially identifiable images or data included in this article.

## Author contributions

HJ: study concept and design HJ, SH, PK and TC: acquisition of samples JS: immunohistochemistry staining and analysis JS, TC and HJ: immunohistochemistry analysis TC and JS: drafting of the manuscript JS: statistical analysis CK: technical and material support MH and TW: study supervision All authors: acquisition, analysis and interpretation of data and critical revision of the manuscript for important intellectual content. All authors contributed to the article and approved the submitted version.

## Supplementary material

Supplementary Figure 1 Secondary Antibody control, peritumoral skin control, positive T17 control. **(A)** Representative confocal images of immunostaining of peritumoral skin using only secondary antibodies anti-goat Alexa Fluor 488 (green), anti-mouse Alexa Fluor 555 (red) and anti-rabbit Alexa Fluor 647 (purple). **(B)** Representative confocal images of immunostaining of peritumoral skin using anti-CD3ϵ (green), anti-TCRδ (red) and anti-IL-17A (purple). **(C)** Representative confocal images of immunostaining of Psoriasis pustulosa using anti-CD3ϵ (green) and anti-IL-17A (purple) as a positive control for IL-17A producing T cells. Dashed lines indicate the border between epidermis **(e)** and dermis. Boxed areas in **(B, C)** are magnified in corresponding second row. Blue: 4′,6-diamidino-2-phenylindole (DAPI). Scale bars represent 40 µm.
