# Supplementary figures and images for: Skin-infiltrating T cells display distinct inflammatory signatures in lichen planus, bullous pemphigoid and pemphigus vulgaris
Source: Front Immunol. 2023 Jun 20;14:1203776. doi: 10.3389/fimmu.2023.1203776 (PMC10321708; doi:10.3389/fimmu.2023.1203776)

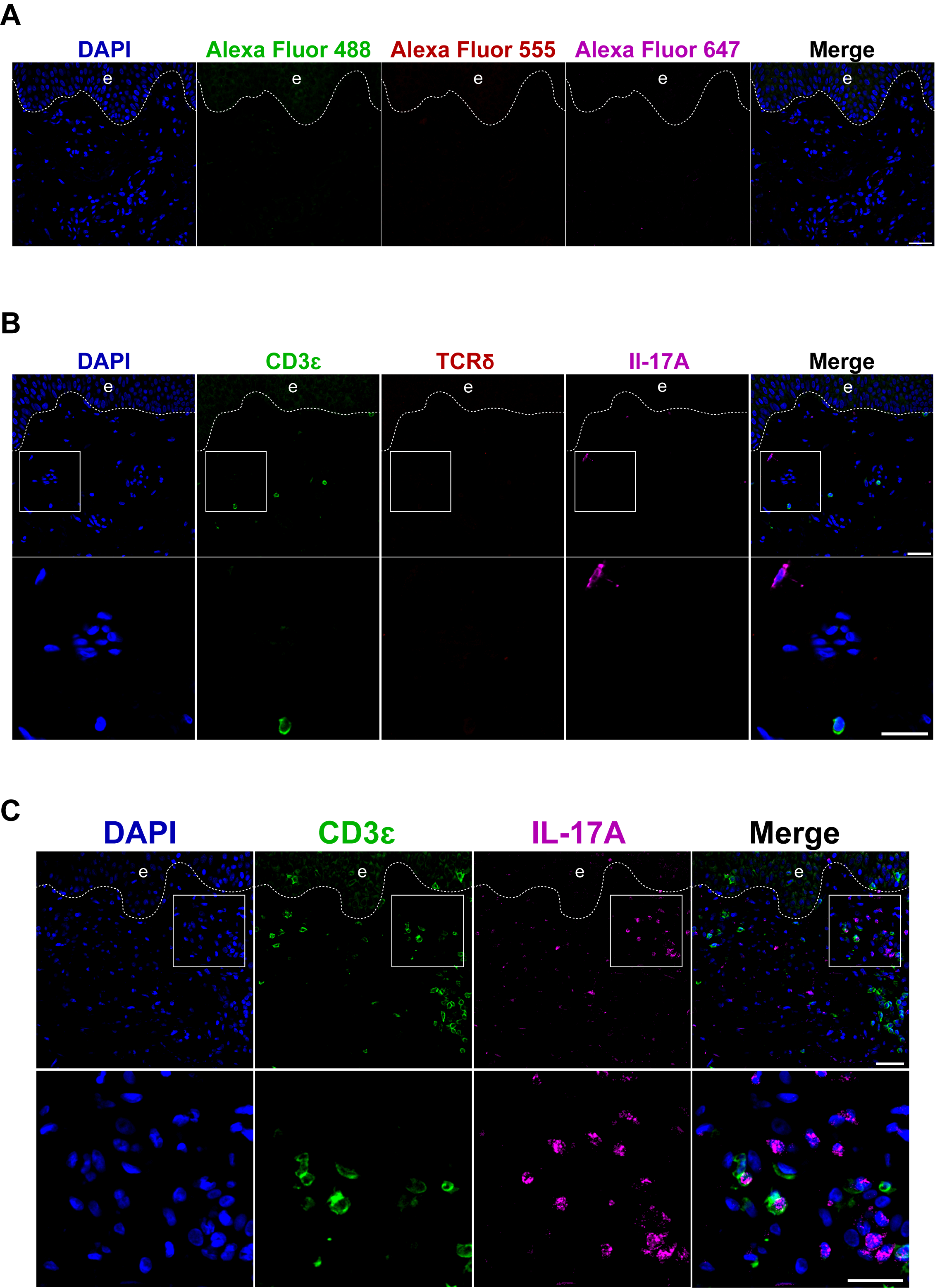

Supplement: Supplementary Figure 1 — Secondary Antibody control, peritumoral skin control, positive T17 control. (A) Representative confocal images of immunostaining of peritumoral skin using only secondary antibodies anti-goat Alexa Fluor 488 (green), anti-mouse Alexa Fluor 555 (red) and anti-rabbit Alexa Fluor 647 (purple). (B) Representative confocal images of immunostaining of peritumoral skin using anti-CD3ϵ (green), anti-TCRδ (red) and anti-IL-17A (purple). (C) Representative confocal images of immunostaining of Psoriasis pustulosa using anti-CD3ϵ (green) and anti-IL-17A (purple) as a positive control for IL-17A producing T cells. Dashed lines indicate the border between epidermis (e) and dermis. Boxed areas in (B, C) are magnified in corresponding second row. Blue: 4′,6-diamidino-2-phenylindole (DAPI). Scale bars represent 40 µm. [file Image_1.tif]
