# Supplementary material for: Skin-infiltrating T cells display distinct inflammatory signatures in lichen planus, bullous pemphigoid and pemphigus vulgaris
Source: Front Immunol. 2023 Jun 20;14:1203776. doi: 10.3389/fimmu.2023.1203776 (PMC10321708; doi:10.3389/fimmu.2023.1203776)
Supplement: Supplementary file 2 [file Table_1.docx]

**Supplementary Table 1**

|  |  | Male | Female |
| --- | --- | --- | --- |
| LP | % of sample | 48 % | 52 % |
|  | Median Age (range) | 66 (34-81) | 56 (26-72) |
| BP | % of sample | 53 % | 47 % |
|  | Median Age (range) | 83,5 (61-89) | 78 (67-88) |
| PV | % of sample | 55 % | 45 % |
|  | Median Age (range) | 64 (50-81) | 60 (55-84) |
