# Supplementary material for: Skin-infiltrating T cells display distinct inflammatory signatures in lichen planus, bullous pemphigoid and pemphigus vulgaris
Source: Front Immunol. 2023 Jun 20;14:1203776. doi: 10.3389/fimmu.2023.1203776 (PMC10321708; doi:10.3389/fimmu.2023.1203776)
Supplement: Supplementary file 3 [file Table_2.docx]

**Supplementary Table 2**

**Supplementary Table 2: Primary and secondary antibodies for immunofluorescence microscopy**

| **Antibody** | **Clone / Conjugate** | **Company** | **Catalog Number** | **Dilution** |
| --- | --- | --- | --- | --- |
| **Primary Antibodies** | | | | |
| **αCD3ε** | SP7 | ThermoFisher | MA1-90582 | 1:150 |
| **αCD15** | MMA | BD Bioscience | 559045 | 1:200 |
| **αCD4** | 4B12 | ThermoFisher | MA5-12259 | 1:50 |
| **αTCRδ** | H-41 | Santa Cruz | sc-100289 | 1:150 |
| **αT-bet** | D6N8B | Cell Signaling Technology | 13232T | 1:150 |
| **αGATA-3** | AF2605 | R&D Systems | AF2605-SP | 1:100 |
| **αIL-17A** | Polyclonal | R&D Systems | AF-317-NA | 1:100 |
| **Secondary Antibodies** | | | | |
| **Donkey αGoat 488** | Alexa Fluor 488 | ThermoFisher | A-11055 | 1:300 |
| **Donkey αMouse 555** | Alexa Flour 555 | Abcam | ab150110 | 1:300 |
| **Donkey αRabbit 647** | Alexa Fluor 647 | Abcam | ab150063 | 1:300 |
